# Verrucous sarcoidosis: a diagnosis to keep in mind

**DOI:** 10.11604/pamj.2020.36.228.21487

**Published:** 2020-07-29

**Authors:** Nadia Nabli, Rima Gammoudi, Amina Aounallah, Badreddine Sriha, Colandane Belajouza, Mohamed Denguezli

**Affiliations:** 1 Department of Dermatology, Farhat Hached University Hospital, Sousse, Tunisia,; 2 Department of Anatomopathology, Farhat Hached University Hospital, Sousse, Tunisia

**Keywords:** Verrucous sarcoidosis, perineum, pulmonary sarcoidosis

## Abstract

Skin manifestations of sarcoidosis occur in up to 30% of cases, and may be the sentinel sign of the disease, with the skin being sometimes exclusively affected. While this may facilitate an early dermatologic diagnosis, heterogeneity in the cutaneous morphologies of sarcoidosis complicates recognition and affirms its reputation as a “great imitator”. Here, we present a case of a verrucous version of sarcoidosis that may be misdiagnosed because it can mimic other inflammatory and neoplastic skin disorders. Although it is a rare variant, its presence should alert clinicians to the likelihood of systemic involvement of cutaneous sarcoidosis.

## Introduction

Skin manifestations of sarcoidosis occur in up to 30% of cases, and may be the sentinel sign of the disease [1]. Cutaneous lesions in sarcoidosis can be divided histologically into specific containing granulomas or reactive nonspecific non-possessing granulomas [2-4]. Sarcoidosis specific lesions are commonly plaques, papules, nodules, scar sarcoidosis or lupus pernio, occurring in a symmetric distribution with a predilection for the face, upper trunk and extremities [3-5]. While this may facilitate an early dermatologic diagnosis, heterogeneity in the cutaneous morphologies of sarcoidosis complicates recognition and affirms its reputation as a “great imitator” [1].

## Patient and observation

**Figure 1:**
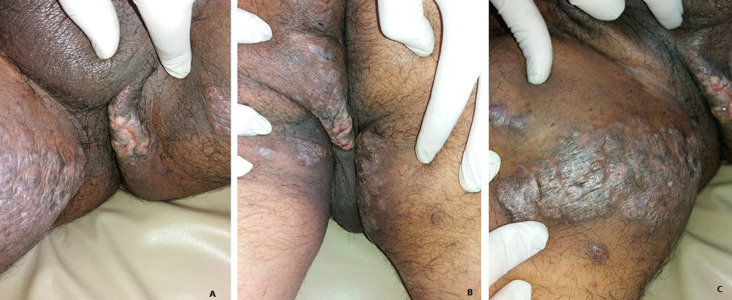
A, B) verrucous plaques with deep fissures on the gluteal region, perineum and inner thighs; (C) close-up view of the verrucous lesion on groin area

**Figure 2:**
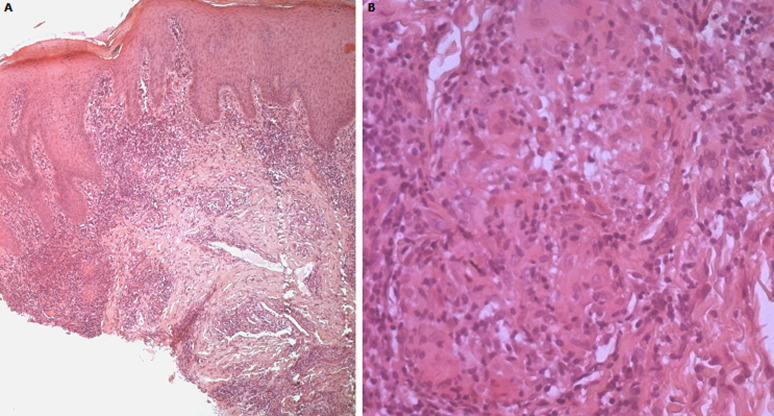
histopathology of verrucous sarcoidosis; A) epidermis shows papillomatous architecture with hyperkeratosis and acanthosis (hematoxylin-eosin stain; original magnification x 40); B) sarcoidal granuloma with multinucleated giant cells surrounded by a sprinkling of lymphocytes, is noted in the dermis (hematoxylin-eosin stain; original magnification x 200)

A 63-year-old man presented with a one-year history of progressive and itchy perineal lesions. On physical examination, the patient had extensive verrucous plaques and nodules of the gluteal region, perineum extending to the inner thighs (Figure 1). Skin biopsy showed multiple small, non-necrotizing, epithelioid cell granulomas in the dermis with hyperkeratosis and acanthosis of the underling epidermis (Figure 2). Special stains and culture of skin tissue failed to indicate any infectious diseases. PCR analyses from skin biopsy failed to detect mycobacterial DNA. Blood investigations showed lymphopenia and elevated serum angiotensin-converting enzyme levels. Endoscopic screening for inflammatory bowel disease was normal. Systemic computed tomography identified hilar and mediastinal lymph nodes associated with subpleural and peri-lymphatic micronodules. Based on the histopathology, radiological findings and the exclusion of other granulomatous etiologies, a final diagnosis of sarcoidosis was made. The patient was managed with topical and general steroids.

## Discussion

Sarcoidosis-specific cutaneous lesions are characterized by variable clinical presentations. This fact makes it difficult for dermatologists to appropriately classify this condition on macroscopic evaluation [1]. Verrucous sarcoidosis is an exceedingly rare variant with only 15 cases previously reported in the English literature [2,4,6]. Including our own case, the majority of patients have been of African descent with an established systemic disease, mostly severe pulmonary involvement [2]. It clinically appears as warty papules and plaques with variable overlying scale, and most commonly affects the lower extremities. Lesions can be mistaken for malignancies or other skin conditions, such as hypertrophic lichen planus and common warts [2,6].

Histologically, these cases showed marked verrucous epidermal hyperplasia with dermal involvement by both typical noncaseating sarcoid granulomas [2]. Only two previous cases of perineal verrucous sarcoidosis have been reported in the literature [2]. In fact, perineal involvement in sarcoidosis is rare and can present as infiltrative plaques, nodules, or swelling in this area. Moreover, those patients must be evaluated for underlying inflammatory bowel disease, which may present similarly or coexist with sarcoidosis. The pathophysiologic basis of verrucous sarcoidosis is not clear. In the present case, the patient complained of pruritus. Therefore, the verrucous epidermal response seen on histopathology could represent an alteration in cutaneous response to persistent mechanical irritation [7]. Furthermore, HPV was identified in a recent case suggesting a possible viral etiology of the verrucous hyperplasia [4]. Previous verrucous sarcoidosis cases describe variable improvement with both topical treatment options and systemic therapies. Relapse after cessation of therapy is common [2].

## Conclusion

Our case highlights that verrucous versions of sarcoidosis may be misdiagnosed because it can mimic other inflammatory and neoplastic skin disorders. Although it is a rare variant, its presence should alert clinicians to the likelihood of systemic involvement of cutaneous sarcoidosis.
